# Egress of HSV-1 capsid requires the interaction of VP26 and a cellular tetraspanin membrane protein

**DOI:** 10.1186/1743-422X-7-156

**Published:** 2010-07-14

**Authors:** Lei Wang, Longding Liu, Yanchun Che, Lichun Wang, Li Jiang, Chenghong Dong, Ying Zhang, Qihan Li

**Affiliations:** 1 Institute Of Medical Biology, Chinese Academy of Medicine Science, Peking Union Medical College, 379# Jiaoling Rd. Kunming 650118 P. R. China

## Abstract

HSV-1 viral capsid maturation and egress from the nucleus constitutes a self-controlled process of interactions between host cytoplasmic membrane proteins and viral capsid proteins. In this study, a member of the tetraspanin superfamily, CTMP-7, was shown to physically interact with HSV-1 protein VP26, and the VP26-CTMP-7 complex was detected both *in vivo *and *in vitro*. The interaction of VP26 with CTMP-7 plays an essential role in normal HSV-1 replication. Additionally, analysis of a recombinant virus HSV-1-UG showed that mutating VP26 resulted in a decreased viral replication rate and in aggregation of viral mutant capsids in the nucleus. Together, our data support the notion that biological events mediated by a VP26 - CTMP-7 interaction aid in viral capsid enveloping and egress from the cell during the HSV-1 infectious process.

## Background

Herpes simplex virus type 1 (HSV-1) is a double-stranded DNA virus with a 152 kb genome that has the capacity to encode more than 80 structural and non-structural viral proteins during its lifecycle in the cell [1]. Its structural proteins generate a dodecahedron protein capsid in the nucleus via their protein-protein interactions [2]. Despite the variety of capsid shapes [3], the basic general structures are composed of interior scaffold polypeptides, principally VP22a, VP21, viral protease VP24 and capsid shell proteins VP5, VP19c, VP23 and VP26 [4]. These proteins tend to assemble into viral capsid hexon and penton structures [5] through a specific mechanism that is not well understood. VP5 is the largest capsid protein and the major component of the capsid shell [6], while VP26 is the smallest capsid protein. VP26 localizes to the surface of penton and hexon capsids as a redundant component [7] and is likely to interact physically with VP5 [8]. Intriguingly, it has been reported that HSV-1 replication and proliferation are not directly affected by the absence of VP26 [9], although the protein is present in viral capsids in high copy numbers (>900/capsid)[10]. However, some studies indicate that the replication rate of viral mutants lacking the VP26-encoding gene UL35 is decreased by 2 - 30 fold in various cell lines[11,12]. Thus, this viral protein potentially has a functionally significant role in the HSV-1 infectious process.

Studies on VP26 functions have demonstrated interactions of VP26 with cytoplasmic dynein light chains RP3 and Tctex 1 when expressed artificially from vectors [13]. Furthermore, VP26 has also been reported to recruit and bind to procapsid in its mature state in an ATP-dependent fashion [14]. Such interactions suggest a possible functional role of VP26 in the transport of viral capsids in the cell[13]; however, this conclusion was not entirely supported by recent studies [15]. Additionally, it is suggested that VP26 on the viral capsid surface might interact with intracellular molecules. Katinka and colleagues used a recombinant HSV-1 virus containing a green fluorescent protein (GFP)-coding sequence fused to UL35 to demonstrate that the fluorescent capsid proteins aggregated in compartments of the cytoplasmic membrane close to the nucleus[16]. The analysis of HSV-1 viral capsid egress from the nucleus indicates that nuclear membrane enveloping is required for these naked capsids to be transported to the perinuclear cisterna [17]. Subsequently, the de-enveloped capsids are wrapped again by organelles such as the Golgi and are gradually moved toward the cytoplasmic membrane and assembled [18]. Together, these data raise the possibility of interactions between protein molecules in viral capsids and components of the cytoplasmic membrane. The significance of host proteins from the cytoplasmic membrane that may interact with viral capsid proteins and become incorporated into the viral envelope in the process of HSV-1 capsid budding merits further investigation.

As demonstrated by cellular and molecular studies, the tetraspanin superfamily member proteins are cellular membrane proteins type III found on the cytoplasmic membrane [19]. Such proteins play essential biological roles with distinctive and differential physiological effects on the viral budding process [20]. In the work described herein, we show that VP26 interacts specifically with tetraspanin superfamily member, cellular tetraspanin membrane protein 7 (CTMP-7) on the cytoplasmic membrane. This interaction may directly affect the enveloping of the HSV-1 capsid shell and contribute to controlling the process and efficiency of virus assembly and egress from the cell.

## Results

### Evidence of VP26 interaction with CTMP-7

**Figure 1:**
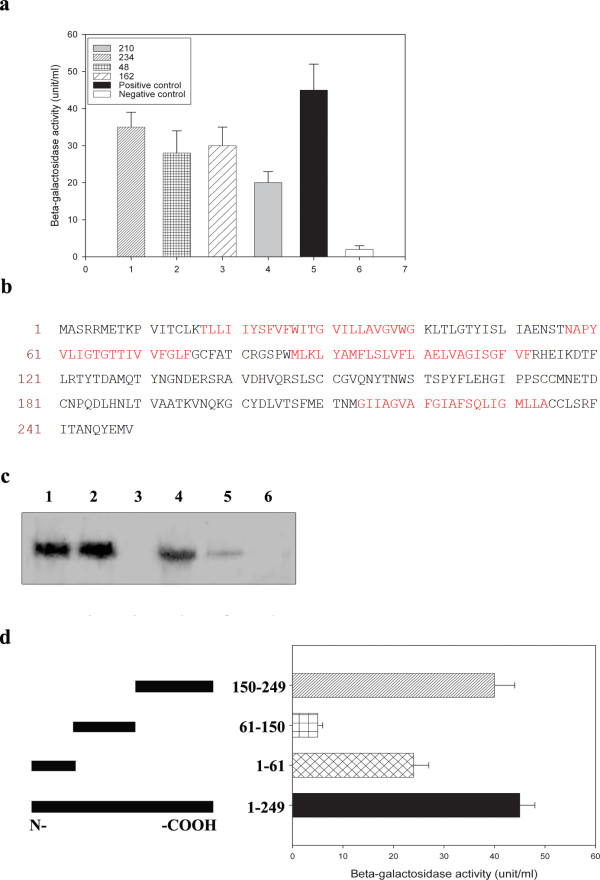
**CTMP-7 is a VP26 interacting protein**. a. Binding assay of the proteins and VP26. The positive control was the fusion yeast containing pGBK-p53 and pACT-LT. The negative control was the fusion yeast containing pGBK-Lam and pACT-LT. b. Amino acid sequence features of CTMP-7. The amino acid sequence of CTMP-7 contains the typical tetraspanin-enriched domain with four transmembrane regions (shown in red) and a topological domain (shown in black). c. Co-immunoprecipitation of the VP26 and CTMP-7 interaction complex and immunoblot by anti-CTMP-7 antibody. Vero cells transfected by VP26 and CTMP gene and labeled with ^35^S-methionine were lysed in RIPA buffer and interacted with anti-VP26 or anti-CTMP antibodies. Lane 1: The immunoprecipitated complexes of cells co-transfected with VP26 and CTMP genes by anti-VP26 antibody; Lane 2: The immunoprecipitated complexes of cells co-transfected with VP26 and CTMP genes by anti-CTMP-7 antibody. Lane 3: The immunoprecipitated complexes of cells transfected with pcDNA mock by anti-VP26 antibody; and Lane 4: The immunoprecipitated complexes of cells transfected with pcDNA mock by anti-CTMP-7 antibody. Lane 5: Control cells lysate; Lane 6: Negative control with normal mouse IgG. d. Mapping the region of CTMP-7 interaction with VP26. The plasmids encoding CTMP-7 amino acid residues 1-61, 61-150 and 150-249 were constructed and transfected into yeast Y187. These transfected Y187 clones were fused with AH109 transfected with pGBK-VP26. These fused clones were identified on QDO plates and their β-galactosidase activity was analyzed.

Five proteins interacting with VP26 were identified by yeast two-hybrid analysis using VP26 as the bait protein and a human embryonic lung mRNA library as the target (Supplementary Table 1). These interactions were further confirmed by a β-gal activity assay (Fig.1a). One of the VP26 interacting proteins is a cellular membrane protein type III and is a homologue of tetraspanin family member cellular tetraspanin membrane protein 7 (CTMP-7, GenBank accession number is NP_004606)). CTMP-7 is composed of 249 amino acid residues (28 kDa) and includes a typical tetraspanin-enriched domain (TEM) (Fig. 1b). We further confirmed binding of CTMP-7 to VP26 in HSV-1 infected human embryonic lung fibroblasts by co-immunoprecipitation with anti-CTMP-7 antibody and anti-VP26 antibody (Fig. 1c). Analysis of the CTMP-7 - VP26 interaction by β-gal activity assay suggested that the interaction involved the C-terminal portion of CTMP-7 (Fig.1d).

**Table 1:** Oligonucleotides used in this study

*Oligonucleotide*	Sequence(5'-3'
CMTP-7-fwd	ATGGAGACCAAACCTGTG
CMTP-7-rev	ACACCATCTCATACTGATTG
VP26-fwd	CGTCCCGCAATTTCACCG
VP26-rev	GGGCGTCGAAGGTTCTCG
UL35-N-fwd	ATGGCCGTCCCGCAATT
UL35-N-rev	CACGGCCCCTTGGGT
UL35-C-fwd	CGGGAGTTTCTCCGCGG
UL35-C-rev	CGAAGGTTCTCGAACGAC

### Expression and distribution of CTMP-7 in normal and HSV-1 infected cells

**Figure 2:**
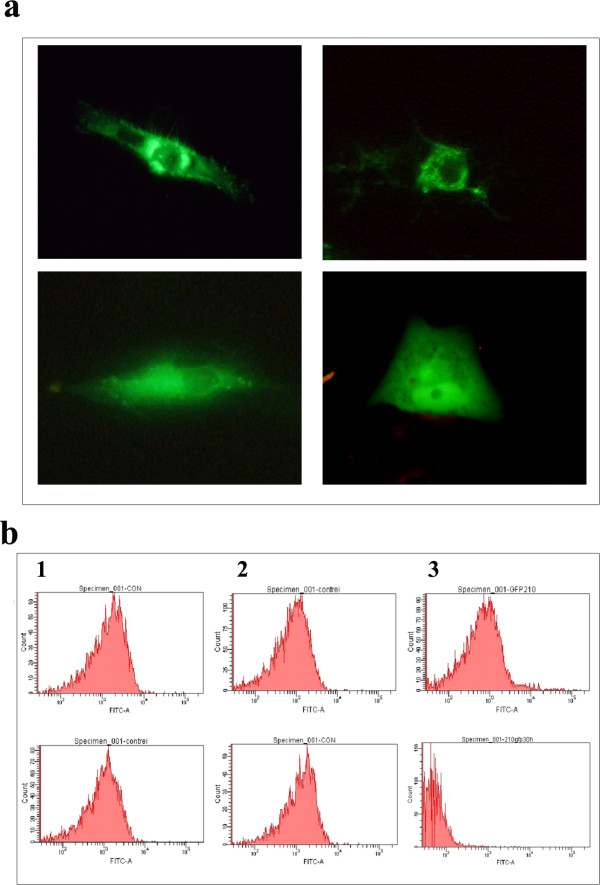
**Intracellular analysis of CTMP-7 in HSV-1 infected cells**. Vero cells were transiently transfected with plasmid pGFP-CTMP or pGFP-N followed by HSV-1 infection at 1 MOI, and then observed at 24 h post transfection by fluorescence microscopy without fixation. At 30 h post-infection, the cells were trypsinized, washed in PBS and analyzed by flow cytometry. a. Observation of Vero cells transfected with pGFP-CTMP or pGFP-N and infected by HSV1 at 24 h post-infection under fluorescence microscope. Upper row: Cells transfected with pGFP-CTMP and infected by HSV-I. Lower row: Cells transfected with pGFP-N and infected by HSV-I. b. Cytometric analysis of Vero cells transfected with pGFP-CTMP and pGFP-N followed by HSV-1 infection or not at 30 h post-infection. Column 1: Cells transfected with pGFP-N, upper - cells HSV-I infection free; lower - cells at 30 h post-infection. Column 2: Control cells transfected with pGFP-CTMP with HSV1 infection free, upper is cell at the same time point to 16 h post-infection of infective example, lower is cells at the same time point to 30 h post-infection of infective example. Column 3: Cells transfected with pGFP-CTMP and followed by HSV1 infection, upper is cell at 16 h post-infection; down is cell at 30 h post-infection.

The fusion protein of CTMP-7 and GFP encoded by eukaryotic expression vector pGFP-CTMP was expressed by transient transfection in Vero cells. Visualization of the expressed CTMP-7-GFP fusion protein in normal cells revealed aggregation in punctate spots on the nuclear and cytoplasmic membranes similar to the pattern exhibited by other tetraspanin family members (Fig.2a, left at upper)[21]. In contrast, the fluorescent punctate distribution was altered drastically in HSV-1 infected Vero cells expressing the CTMP-7-GFP fusion protein. Bright punctate spots were distributed from the nuclear membrane to the cytoplasm at 24 h post-infection (Fig.2a, right at upper). Compared with control cells expressing fluorescent GFP protein where no significant alteration was visualized (Fig 2a down row). Flow cytometric analysis showed an obvious decrease of fluorescence intensity in cells expressing tetraspanin-GFP fusion protein at 30 h post HSV-1 infection (Fig.2b).

### CTMP-7 molecules are present in purified HSV-1 virions

**Figure 3:**
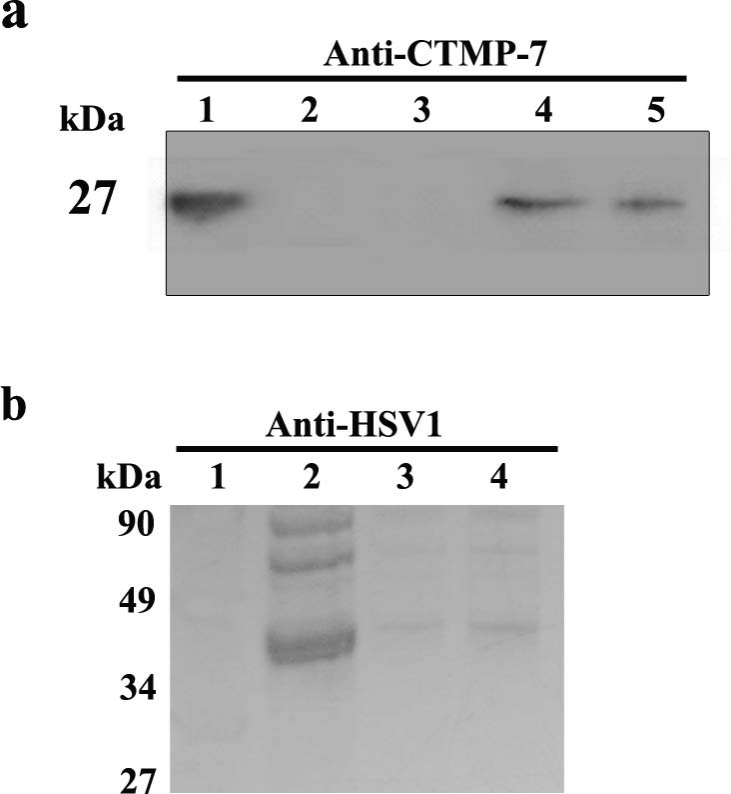
**Western blot of CTMP-7 molecules in purified HSV-1 viral particles**. a. Immunoblot showing the distribution of cellular CTMP-7 protein in HSV-1 infection. KMB-17 cells infected with HSV-1 (1 MOI) or uninfected were collected at 48 h post-infection and centrifuged at 10,000 rpm for 10 min at 4°C to separate debris and supernatant. The supernatant from infected cells was concentrated to 1/50 by centrifugation at 40,000 rpm for 4 hours at 4°C, re-suspended, and separated via a sucrose density gradient centrifugation. The fraction with highest titer was used in immunoblot. 1: Control cells debris; 2: Control cells supernatant; 3: The supernatant from infected cells; 4: The pellet from infected cells; 5: Purified virions from sucrose gradient centrifugation. The antibody in this immunoblot is mouse polyclonal antiserum against CTMP-7. b. Western blot analysis with anti-HSV1 antibody. The purified virus virion was electrophoresed by 10% SDS-PAGE, transferred onto nitrocellulose membranes, and used for Western blotting analysis with anti-HSV-1 antisera specific for the virus protein at 1:500 dilution. Signals were detected by an ECL system (Pierce). 1: Control cells debris; 2: Purified virions from sucrose gradient centrifugation; 3: The supernatant from infected cells; 4: The pellet from infected cells.

Upon observing that fluorescently tagged CTMP-7 molecules disappeared from nuclear and cytoplasmic membranes in HSV-1 infected cells, we performed a Western blot assay to detect CTMP-7 in infected cell debris, infected cell culture supernatant, and in concentrated, purified virus isolated by sucrose density gradient centrifugation. Intriguingly, CTMP-7 protein was detected in infected cell debris, and a low level of CTMP-7 protein was detected in purified viral particles showing 27 kDa band in Western blot by antibody against-CTMP-7(Fig.3a), and the other Western blot analysis with anti-HSV1 antibody confirmed the band did not represent a viral component (Fig.3b). Since the purified viral particles were harvested intact from the gradient, this result suggests that capsid enveloping and entry into viral assembly may be attributed to CTMP-7 binding of VP26.

### Inhibition of CTMP-7 expression decreases viral replication

**Figure 4:**
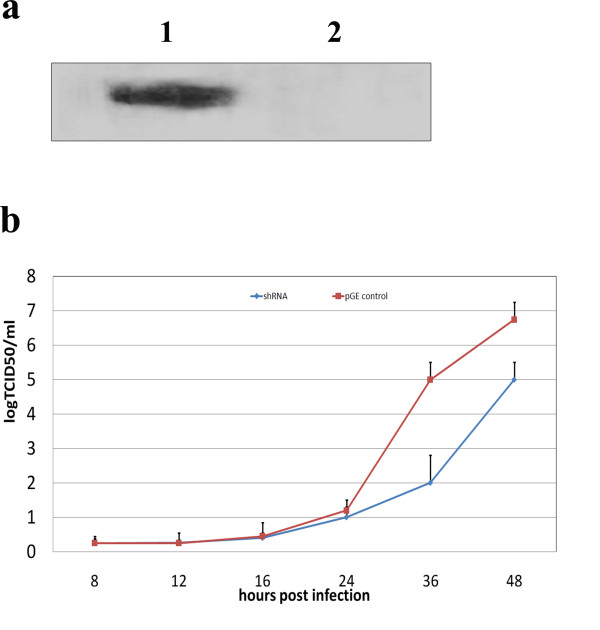
**Inhibition of CTMP-7 in cells by RNAi delayed the proliferation of HSV-1**. a. Expression of CTMP-7 in KMB-17 cells was inhibited by pGE-CTMP containing siRNA targeted specifically to CTMP-7 mRNA. Cells transfected by pGE-CTMP or pGE-1 were harvested after 48 h, and lysates were analyzed by Western blotting using antisera raised in mouse immunized with CTMP-7. 1: Cells transfected with pGE-1; 2: Cells transfected by pGE-CTMP. b. Inhibition of CTMP-7 expression by siRNA reduced HSV-1 proliferation in fibroblasts. Growth curves of HSV-1 in KMB-17 cells transfected with pGE-CTMP were produced and analyzed. KMB-17 cells transfected with pGE-1, pGE-CTMP plasmid were infected at an MOI of 1 with HSV-1, and incubated at 37°C. At the indicated times post-infection, samples of cell supernatant were removed and the viral titer was determined by microtitrating assay. *n *= 3 for all time points. Error bars represent the standard error of the mean.

It has been demonstrated that proliferation and replication of an HSV-1 mutant lacking the VP26 encoding gene were significantly delayed compared to wild-type virus [11]. If the potential interaction of VP26 with CTMP-7 is significant, similar effects on proliferation and replication should be observed in the absence of CTMP-7. In order to investigate the hypothesized importance of CTMP-7, the plasmid pGE-CTMP was transfected into human embryonic lung fibroblast cells (KMB-17). Plasmid pGE-CTMP expresses siRNAs directed against the 3'-UTR and coding sequences of the CTMP-7 mRNA. The knockdown effects of pGE-CTMP were confirmed by examining CTMP-7 protein levels in a Western blot (Fig.4a). Compared to cells transfected with the control plasmid pGE-Neg, viral growth was significantly decreased 24-36 h post HSV-1 infection in cells transfected with the siRNA-expressing pGE-CTMP plasmid (Fig. 4b). This result implies that CTMP-7 is required for efficient HSV-1 replication and proliferation.

### Reduction of CTMP-7 decreases egress of HSV-1 particles

**Figure 5:**
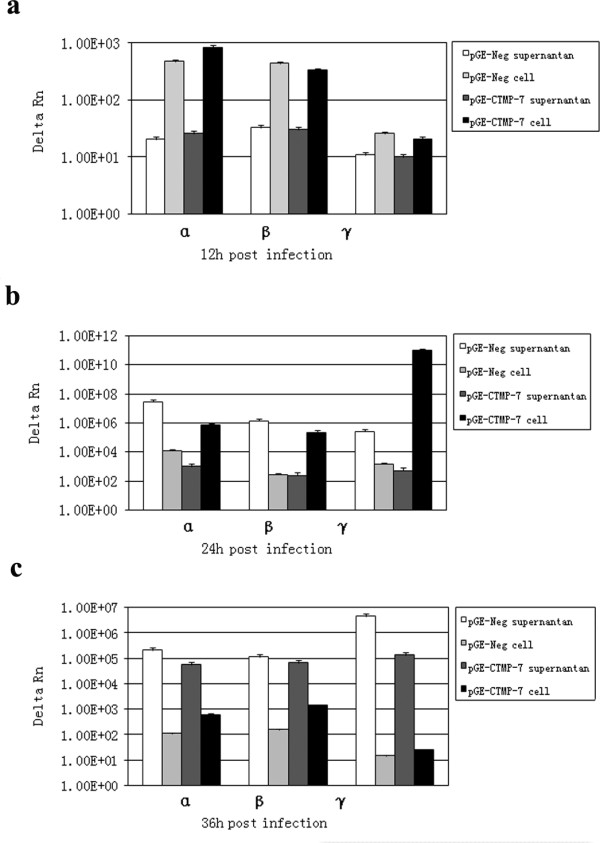
**Reduction of CTMP-7 in cells by RNAi decreased the egress of HSV-1**. KMB-17 cells transfected with pGE-Neg or pGE-CTMP plasmid were infected at an MOI of 1 with HSV-1 and incubated at 37°C. Viral loads (shown as delta Rn of virus DNA replication) of HSV-1 in KMB-17 cells were detected and analyzed by real-time PCR. At the indicated times post infection, samples of cell transfected and infected were harvested and extracted for further real-time PCR. The protocol is described in Material and Method. a. Viral loads shown with the copy of α-4, tk and gC genes of HSV1 at 12 hours post-infection in cells with CTMP-7 expression being inhibited. b. Viral loads shown with the copy of α-4, tk and gC genes of HSV1 at 24 hours post-infection in cells with CTMP-7 expression being inhibited. c. Viral loads shown with the copy of α-4, tk and gC genes of HSV1 at 36 hours post-infection in cells with CTMP-7 expression being inhibited.

VP26 is located on the viral capsid surface, while its functionally interacting molecule CTMP-7 is a trans-membrane protein in the cytoplasmic membrane. We hypothesized that the effect of decreased CTMP-7 levels on viral replication most likely takes place during the egress of HSV-1 particles. Therefore, the egress of viral particles was further examined in cells with CTMP-7 expression inhibited by RNAi. In order to accurately detect the variations of virus numbers in infected cells, specific primers were designed to target HSV-1 α-4, TK and gC genes. These primers were employed to determine cellular or extracellular viral loads at different infectious stages by using real-time quantitative PCR. The results showed that the viral loads 12 h post-infection were similar in cells transfected with pGE-CTMP and pGE-Neg (Fig.5a). At 24 h post-infection, the viral load in control cell supernatants was significantly higher than the intracellular viral load. In contrast, the intracellular viral load in cells with suppressed CTMP-7 expression was much higher than that in the supernatant (Fig.5b). By 36 h post-infection of pGE-CTMP-transfected cells, the viral load was higher in the supernatant than in the cells, although there were still some differences in the virus levels compared to the control cells (see Fig.5c).

### Inhibition of CTMP-7 results in HSV-1 capsids aggregating in the cell

**Figure 6:**
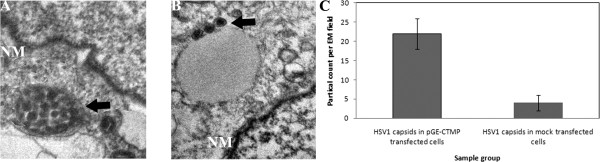
**Morphological analysis on HSV-1 capsids aggregating in the cell with CTMP-7 expression inhibited (X30, 000)**. KMB-17 cells transfected with pGE-1 or pGE-CTMP plasmid were infected at an MOI of 1 with HSV-1 and incubated at 37°C. At 48 hour post-infection, samples of cell were harvested, and the enveloping process of viral capsids was observed by electron microscopy. a. HSV-1 capsids were observed aggregating in the area closing to nuclear membrane (NM) of cells transfected by pGE-CTMP. b. HSV-1 capsids were localized separately in nuclear and cytoplasma of cells transfected by pGE-1 mock plasmid. c. HSV-1 capsids were counted in difference EM fields. *n *= 3 for all fields. Error bars represent the standard error of the mean.

The previous experiments demonstrated that viral replication is similarly affected, i.e., reduced, in cells infected by HSV-1 viruses in the absence of either VP26 or CTMP-7. The detailed mechanism of how these proteins affect HSV-1 replication remains unclear, although the available evidence suggests that the absence of CTMP-7 affects the egress of viral particles. In attempt to determine the mechanism, a morphological analysis was performed using electron microscopy to observe the enveloping process of viral capsids. The HSV-1 capsids were observed aggregating in cells in which CTMP-7 expression was inhibited by RNAi (Fig.6a). However, in control cells the capsids were localized separately to the cytoplasma (Fig.6b). This observation suggests preliminarily that VP26 and CTMP-7 may contribute to the egress of viral particles by contributing to the process of enveloping of viral capsids.

### A VP26 mutant causes viral capsid aggregation in the cell and leads to a decreased viral replication rate

**Figure 7:**
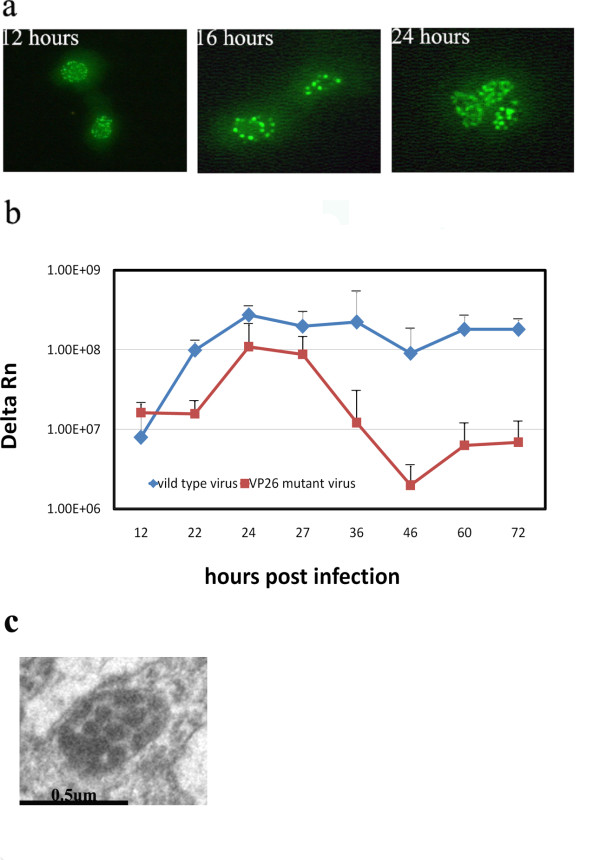
**VP26 mutant virus shows a delayed proliferation**. The recombinant virus HSV1-UG with a GFP-UL35 fusion gene was used to infect Vero or KMB-17 cells at 1 MOI. Punctated fluorescence spots were observed in the nucleus during infection, which is similar to the recombinant HSV1 with GFP-Vp16 fusion gene ([31]). At 12, 22, 24, 27, 36, 46, 60 and 72 h post-infection, samples of cell infected by HSV1-UG were collected and measured by real-time PCR with specific primers against α-4 gene. n = 3 for all time points. Error bars represent the standard error of the mean. Meanwhile, the KMB-17 cells infected by HSV1-UG were fixed with 5% glutaraldehyde and observed under the electron microscope. a. The Vero cells infected by VP26 mutant HSV1-UG were observed under fluorescence microscope at 12, 16 and 24 h. b. Growth curve of HSV1-UG compared with that of wild type HSV-1 in Vero cells. c. Electro-microscope observation of cells infected by HSV1-UG(X30, 000).

In order to further investigate the biological events in the viral enveloping process generated by the interactions of VP26 and cellular CTMP-7, we constructed a viral mutant, HSV-1-UG, with a GFP-coding sequence fused to the UL35 gene. In Vero cells infected with HSV-1-UG, a mutant VP26 was expressed and distributed in fluorescent punctate spots throughout the infected cells and maintained this pattern until 24 h post-infection (see Fig.7a). Similar observations were found in the mutant HSV-1 infected HeLa cells and neuroma SH-5YSY cells (data not shown). However, the kinetic growth rate of this viral mutant was significantly lower than that of wild-type HSV-1. Interestingly, when the HSV-1-UG mutant was used to infect normal human embryonic lung fibroblast KMB-17 cells, aggregation of viral capsids in the cells was observed (Fig.7b). These data support the previous work performed in our laboratory.

## Discussion

The biological events of HSV-1 progeny viral assembly and maturation during infection constitute a self-controlled process of virion enveloping after assembly of the capsid [22]. Extensive examination by electron microscopy has shown that the HSV-1 capsid shell is incorporated in the nucleus and subsequently transported toward the interior nuclear membrane by an unknown mechanism; this process leads to the capsid being enveloped by the nuclear bi-layer membranes. The interior membrane is considered the first viral envelope and the exterior membrane facilitates egress of the enveloped virion from the nucleus via the cis-network and remains in the cisterna of perinuclear space. The virion is then transported from cisterna to organelles like Golgi in the cytoplasm where the first incorporated envelope is assumed to de-envelope [23]. The naked capsids then enter into the Golgi secretory pathway and are enveloped via binding to the cytoplasmic membrane, leading to exocytosis and egress of the whole assembled virus from the cell [24]. Although this putative model is supported by some studies, others suggest another model that presumes the redundancy of the second enveloping by deducing that the viral capsids are directly transported to the Golgi secretory pathway and out of the cell by exocytosis [25]. Certainly, no matter which of the two models is more plausible, both basically involve the premise that the enveloping of viral capsids requires interactions of capsid components with the cytoplasmic membrane [26]. In this context, the efficiency and rate of viral capsid enveloping are likely to contribute to the control of the efficiency and rate of viral replication.

The studies of HSV-1 VP26 demonstrate that this protein is similar to other viral proteins that are not absolutely required for viral replication [9]. However, from the viewpoint of evolution it does not follow that VP26 would maintain high copy numbers but not be functional in capsids. Thus, it is not surprising that the interactions of VP26 with several cellular proteins in the yeast two-hybrid assay were observed. We also confirmed that VP26 interacted physically with CTMP-7, a tetraspanin superfamily member consisting of typical transmembrane structures. Furthermore, as shown in co-immunoprecipitation assays performed on HSV-1 infected cells, the VP26-CTMP-7 complex was detected equally in infected cells by either anti-VP26 antibody or CTMP-7 antibody. These results allow us to hypothesize a potential biological significance of VP26 binding to CTMP-7 for the HSV-1 infectious process. It may seem unlikely that VP26 would mediate a series of functions by binding to the C-terminus of CTMP-7, which only contains 12 amino acid residues. However, the reported finding that the HTLV-1 Gag protein could bind to a 5 amino acid domain of CD82 (another tetraspanin member) and use this interaction to enter the host cell suggests the VP26-CTMP-7 interaction is possible [27].

The distribution of CTMP-7 in normal fibroblasts visualized by fusion to a GFP protein showed that it was a typical cellular membrane protein diffused on the surface of the nuclear and cytoplasmic membranes (Fig. 2a). This distribution was altered remarkably by fluorescent particles in diffused punctate patterns moving away from the cytoplasma at 24 h in HSV-I infection, about the time a replication cycle of this virus would be completed (Fig.2a). With the viral infection extended to 30 h, the GFP-fused CTMP-7 molecules on infected cytoplasmic membrane gradually tended to disappear as compared to that on non-infected cytoplasmic membrane compared with GFP control cells in cytometric analysis (Fig. 2b). Additionally, CTMP-7 molecules were visualized in purified HSV-1 virions in Western blot by antibody against CTMP-7, and Western blot of purified virion proteins by anti-HSV1 antibody confirmed this obversation (Fig.3a,b), corresponding to the disappearance of CTMP-7 from the cells during the progress of viral infectivity (Fig.3a). It remains unclear whether CTMP-7 packaged in virions would function in the initial process of viral infection. The data described herein strongly support the hypothesis that the interaction of VP26 with CTMP-7 plays an essential role in accomplishing normal viral replication. Based upon this hypothesis, our study examined the effects of inhibiting CTMP-7 expression by two specific RNAi molecules produced by the pGE-CTMP vector in infected cells. We provided further evidence of the functional role of CTMP-7 by observing a significant decrease in the HSV-1 replication rate when CTMP-7 expression was inhibited (Fig.4-b). Moreover, as shown by real-time quantitative PCR analysis, delayed viral enveloping likely leads to a reduced number of mature virions egressing from the infected cell as compared to that of normal cells in the same period of time (Fig. 5a, b, c). The detection of α, β, and γ gene copy numbers in infected cell supernatant and precipitation of the control and experimental groups revealed no remarkable correlation between decreasing viral replication rate and gene transcription and replication processes in cells with CTMP-7 RNAi treatment. Instead, this observed decrease in viral replication is probably attributed to inhibition of capsid enveloping and egress of viral particles. As further demonstrated by our electron-microscopic observations, in the absence of CTMP-7 the capsid enveloping was stopped temporarily and capsids were aggregated in the nucleus (Fig. 6a, b). Nevertheless, our experiments indicated that the number of virions egressing was increased again at later infectious stages (36 h post-infection) regardless of the initial impact of CTMP-7 absence on capsid enveloping (Fig.5-c). This observation suggests that HSV-1 may have other compensating strategies to overcome the delay of viral infectious cycles due to capsids aggregating in the nucleus in the absence of CTMP-7.

The tetraspanin superfamily member proteins are broad transmembrane proteins with physiological functions associated with many signal transduction pathways and pathological processes of many infectious diseases [28]. The infectious and proliferative processes of some RNA viruses such as HIV, HTLV-1 and HCV are proposed to associate with tetraspanin molecules as well [27,29,30]. The findings in this study will enable better understanding of the biological mechanisms of HSV-1 viral capsid enveloping mediated by interactions of CTMP-7 and VP26.

## Conclusion

We demonstrated the interaction of VP26 with cellular CTMP-7 in our *in vivo *and *in vitro *experiments. In addition, analysis of recombinant virus HSV-1-UG showed that mutating VP26 resulted in a decreased viral replication rate and aggregation of viral mutant capsids in the nucleus. Together, our data lend support to the conclusion that the biological events mediated by VP26 interacting with CTMP-7 aid in the viral capsid enveloping and egress from the cell during the HSV-1 infectious process.

## Methods

### Cells and Virus

KMB17 human embryo fibroblasts (passage 27, Institute of Medical Biology, CAMS), Vero cells (passage 219, ATCC) and Hela cells were grown in Dulbecco's Modified Eagle's Medium (DMEM) (Gibco, Grand Island, NY, USA) supplemented with 50 mmol/L L-glutamine (Sigma, St. Louis, MO, USA) and 10% (v/v) of fetal bovine serum (FBS, Gibco) and incubated in 5% CO_2 _at 37°C. Chinese hamster ovary (CHO) cells were grown in complete Ham's F12 media containing 5% fetal calf serum under 5% CO_2 _at 37°C. Herpes simplex virus 1 (F strain obtained from the Institute of Virology, Beijing) was grown in Vero cells and titered in the same cells with a micro-titration assay.

### Plasmid Construction

Vectors pcDNA (Invitrogen, Grand Island, NY, USA), pGFP-N (Invitrogen), pGE-1, pGE-Neg (Stratagene), pGBK-T7 (Clontech, Palo Alto, CA, USA) and pBV220 (China CDC) were produced and purified according to standard protocols for further plasmid construction. The primers used to obtain the sequences of the UL35 gene and cellular tetraspanin membrane protein 7 (CTMP-7) were as Table. 1. pGBK-Vp26 was constructed with pGBK-T7 and the encoding sequence of UL35. pcDNA-UL35 was constructed by pcDNA-3 and the encoding sequence of UL35. pGFP- CTMP was constructed with pGFP-N and the encoding sequence of CTMP-7. pcDNA-CTMP was constructed by pcDNA-3 and the encoding sequence of CTMP.

### Yeast two-hybrid screen and β-galactosidase assay

The cDNA of UL35 was cloned into pGBK-VP26 as a Gal4 DNA-binding domain fusion. This construct was used to screen a pre-transformed human liver cDNA library (BD Biosciences Clontech). Approximately 104 transformants were screened according to the manufacturer's protocol. The positive clones were identified twice on synthetic dropout agar plates lacking leucine, tryptophan, histidine and adenine (QDO) and were cloned and sequenced. The β-galactosidase assay was performed to compare the relative strength of interaction between VP26 protein and selected proteins with the substrate o-nitrophenyl-β-D-galactosidase (ONPG, Sigma). β-galactosidase units were calculated using the formula: β-galactosidase units = 1000 × A420/(t × V × A600). T = elapsed time of incubation (min); V = 0.1 ml × concentration factor; A600 = A600 of 1 ml of culture.

### Co-immunoprecipitation of VP26 and CTMP-7 in vivo

Co-immunoprecipitation of VP26 and CTMP-7 was performed according to a standard protocol. Vero cells grown in DMEM with 5% FBS to 90% confluence in six-well plate were washed twice with serum-free DMEM and were transfected with 2 ug/well of pcDNA-UL35Invitrogen) After transfection, the cells were recovered in DMEM supplemented with 5% FBS for 24 h. Subsequently, the transfected cells were incubated in DMEM at 37°C and maintained in the same media for more than 8 h. The cells were rinsed twice with PBS and scraped in 100 μl of modified RIPA buffer (150 mmol/L NaCl, 1% Brij-96, 0.5% deoxycholic acid, 50 mmol/L Tris-HCl pH 7.5), and freeze-thawed 3 times. After centrifugation at 12,500 rpm for 10 min at 4°C, the supernatant was incubated with an anti-VP26 monoclonal antibody (Upstate Biotechnology) or with an anti-CTMP-7 (VP26 interacting protein) polyclonal antibody in RIPA buffer at 37°C for 1 h. The A protein-Sepharose 4B (Sigma) was added for further incubation at 4°C for 1 h. After washing 3 times with washing buffer (50 mmol/L, 1% Brij-96, 0.1% SDS, 50 mmol/L tris-HCL, pH7.5) and centrifugation as above, the A protein-Sepharose 4B absorbed immune complex pellet was incubated in SDS sample buffer (2% SDS, 62.5 mmol/L Tris, 10% glycerol, 2% 2-mercaptoethanol pH 6.8) at 100°C for 5 min. The supernatant was subjected to SDS-PAGE followed by electrophoresis and transferred to NC membrane. Finally, the membrane was used for further Immunoblot by anti-CTMP antibody.

### Fluorescence Detection of CTMP-7

Vero cells plated in 6-well plates at 90% confluence in DMEM supplemented with 10% FBS were transiently transfected with control plasmid pGFP-N and expression plasmids pGFP-CTMP using Lipofectamine Plus reagent (Invitrogen). All cell samples were viewed under a fluorescence microscope 36 h after transfection. For analysis of the effect of CTMP-7 in the HSV-1 infected cell, KMB-17 cells pre-transfected with pGFP-CTMP was infected with HSV-1 at 0.2 multiplicity of infection (MOI). At 12, 24, 36, 48, 60 and 72 h post-infection, the cells were trypsinized, washed in PBS and analyzed by flow cytometer (Facs Canto II, BD).

### Virus purification

Herpes simplex virus 1 harvested from Vero cells was first centrifuged at 3,500 rpm for 20 min. 1M ZnAc_2 _was then added to the resulting supernatant to yield ZnAC_2 _concentration of 20 mM. The mixture was incubated at 4°C for 30 min and then centrifuged at 10,000 rpm for 30 min to collect the precipitate which contained HSV-1. HSV-1 was further purified by sucrose density gradient centrifugation. Briefly, a 0.5 mL sample was applied to a 20% to 50% linear sucrose gradient (containing 20 mmol/L Tris-HCl plus 150 mmol/L NaCl, 10 mmol/L MgCl_2_, 1% NP-40) prepared in a 5 mL centrifuge tube. Gradients were centrifuged for 5 h at 45,000 rpm at 4°C. The gradient was fractionated, and identified by OD280nm.

### Viral growth kinetics analysis

A one-step growth curve was produced to determine whether CTMP-7 had an effect on HSV-1 virus proliferation. KMB-17 cells were infected with HSV-1 virus at an MOI of 1 in the presence or absence of CTMP-7. The infected cell supernatants were harvested at the indicated times and the titer was determined by microtitration.

### RNA interference (RNAi) of CTMP-7 gene expression

RNAi-mediated reduction of the CTMP-7 gene expression in KMB-17 cells was performed with a specific double stranded small hairpin RNA (shRNA) fragment against the gene encoding CTMP-7. Two siRNA fragments were against the nucleotides 179 to 208 and 553 to 582 of the CTMP-7 gene respectively:

5'-GGATCCCG**CAGGCCAAAGACAACAATAGTGGTGCCAG**TCAAGAG**CTGGCACCACTATTGTTGTCTTTGGCCTG**tcgtcagctcgtgccgtaag

**TGAAACTAGTTACCAGATCATAACAACCC**TCAAGAG**GGTTGTTATGATCTGGTAACTAGTTTCA**TTTTTTCTAGA-3' was inserted into pGE-1 to be as pGE-CTMP. In addition, a scrambled interfering RNA was used as the negative control. The sequences were produced with the pGE-1 vector. Beginning twenty-four hours after transfection, cells were harvested at the designated time points (usually 0, 6, 12, 24, 48, 72 and 96 h), and the expression of CTMP-7 protein was detected in KMB-17 cells by Western blot with the antibody against CTMP-7.

### Quantitative real-time PCR

For each sample, 500 ng of the DNA was used after purification from clarified supernatant of HSV-1 infected cells using the QiaAmp DNA Blood Mini Kit (Qiagen) as per the manufacturer's protocol. As a set of internal standards, the HSV-1 virus was serially diluted to known concentrations in the range of 10^1 ^to 10^7 ^molecules per μL with the total concentration of DNA in each adjusted to 50 μg/mL with salmon sperm DNA. PCR reactions were prepared using the SYBGRN PCR kit (TOYOBO) in 20 μL volumes. Primers for amplification of a-4 (amplicon size: 80 bp), tk (amplicon size:111 bp) and gC gene (amplicon size:108 bp) were 5'- GGAGACGTCGTCACGGCCGG -3' and 5'- TCGTTGCCGTCGTCGTCCTC -3', 5'- AGGCATGCCCATTGTTATCTG -3' and 5'- GAGACAATCGCGAACATCTAC -3', 5'- ATTCCACCCGCATGGAGTTC -3' and 5'- CGGTGATGTTCGTCAGGACC -3', respectively. Reactions were run in a 96-well format in an ABI Prism 7500 with the following conditions: 95°C for 15 min, followed by 95°C for 15 s, 60°C for 1 min and repeated for 30 cycles. Cts were determined as the first cycle where fluorescence was 10 times background.

### Western blot

Western blot with the antibody produced by the expressed CTMP-7 protein was performed to detect the expression of CTMP-7 according to standard protocol. As an internal control, mouse monoclonal anti-β-actin antibody (diluted 1:200; Santa Cruz Biotechnology, Santa Cruz, CA) was used. The secondary antibody, anti-mouse IgG, was purchased from Sigma. Nitroblue tetrazolium (NBT; Sigma) was used as the substrate to detect reactivity.

### Construction of VP26-EGFP fusion protein recombinant virus

Briefly, cells were infected with HSV-1 (MOI = 0.1) and transfected at 30 min post-infection with pGFP-UL35N-GFP-UL35C. The infection was allowed to proceed for 2 to 3 days before cells were harvested. Confirmation of the presence of GFP was obtained by performing PCR with primers that flank the transcriptional start site of the UL35 gene. Products of 320 bp and 1123 bp were amplified from recombinant viruses and non-recombinant viruses, respectively. These viruses were then subjected to two rounds of plaque purification.

## Abbreviations

HSV-1: Herpes simplex virus type 1; GFP: Green fluorescent protein; CTMP-7: Cellular tetraspanin membrane protein 7; TEM: Tetraspanin-enriched domain; DMEM: Dulbecco's Modified Eagle's Medium; FBS: Fetal bovine serum; ONPG: o-nitrophenyl-β-D-galactosidase

## Competing interests

The authors declare that they have no competing interests.

## Authors' contributions

QL conceived of the study, and participated in its design and coordination, QL and LL revised the manuscript. LW and LL participated in the design of the study and performed analysis, LW, CD and YZ participated in the study. All authors read and approved the final manuscript.
